# Genomic Signal Processing

**DOI:** 10.2174/138920209789177593

**Published:** 2009-09

**Authors:** Edward R Dougherty, Yufei Huang, Seungchan Kim, Xiaodong Cai, Rui Yamaguchi

Genomic Signal Processing (GSP) has been defined as the analysis, processing, and use of genomic signals for gaining biological knowledge and the translation of that knowledge into systems-based applications, where by genomic signals we mean the measurable events, principally the production of mRNA and protein carried out within the cell. Owing to the defining role of DNA in the production of mRNA, the structural characterization of DNA is inevitably a part of GSP and, interestingly, signal processing methods are utilized in understanding DNA structure.

A key goal of translational genomics is to discover families of genes or gene products that can be used to classify disease, thereby leading to molecular-based diagnosis and prognosis. A deeper goal is to characterize genomic and proteomic regulation, thereby leading to a functional understanding of disease and the development of systems-based medical solutions. 

GSP is growing in importance as an ever larger community is recognizing that accomplishing these goals requires various disciplines within or related to signal processing, including pattern recognition, prediction/estimation theory, information theory, dynamical systems, control theory, network modeling, and communication theory. In sum, systems biology and systems medicine demand deep understanding of systems theory. This inevitably entails the theory and methods of signal processing, which have been so successful in areas such as communications, and the related theory pertaining to the characterization and control of dynamical systems, without which one cannot even imagine our contemporary technological society.

The purpose of this special issue is to bring some of the key developments in GSP to the wider genomics community. Owing to its grounding in systems theory and stochastic processes, GSP often requires mathematics beyond the level of that studied in undergraduate electrical engineering, or even undergraduate mathematics and statistics, and, therefore, as originally published in the scientific literature, is not accessible to many researchers in biology and medicine. Because systems biology and systems medicine will, *ipso facto*, have to rely on mathematical systems theory, this dichotomy is a problem that will have to be addressed in the future, from both educational and research perspectives; nonetheless, in a review format it is possible to communicate many of the basic ideas without recourse to the kind of full rigorous mathematical analyses required in original research. 

In this issue, the guest editors have tried to accomplish this aim in two ways: first, by specifying the kinds of issues we would like contributors to address, with the requirement that the mathematical details be kept to a minimum (with references to the relevant literature); and, second, by working with the contributors to achieve the goals of the issue through the review process. 

Although the final scope has been determined by the submissions and acceptances, we believe that a good breadth of subject matter is included in the issue, including the inference, analysis, and control of gene regulatory networks, mass spectrometry for proteomics, sequence analysis, metagenomics, MicroRNA target prediction, clustering, and feature selection and error estimation for classification, where in the cases of classification and clustering the papers pay close attention to performance in the context of small samples, a ubiquitous situation in which many methods in the literature perform poorly.

